# Analysis of the Trajectory of *Drosophila melanogaster* in a Circular Open Field Arena

**DOI:** 10.1371/journal.pone.0001083

**Published:** 2007-10-24

**Authors:** Dan Valente, Ilan Golani, Partha P. Mitra

**Affiliations:** 1 Cold Spring Harbor Laboratory, Cold Spring Harbor, New York, United States of America; 2 Department of Zoology, Faculty of Life Sciences, Tel Aviv University, Ramat Aviv, Tel Aviv, Israel; University of East Piedmont, Italy

## Abstract

**Background:**

Obtaining a complete phenotypic characterization of a freely moving organism is a difficult task, yet such a description is desired in many neuroethological studies. Many metrics currently used in the literature to describe locomotor and exploratory behavior are typically based on average quantities or subjectively chosen spatial and temporal thresholds. All of these measures are relatively coarse-grained in the time domain. It is advantageous, however, to employ metrics based on the entire trajectory that an organism takes while exploring its environment.

**Methodology/Principal Findings:**

To characterize the locomotor behavior of *Drosophila melanogaster*, we used a video tracking system to record the trajectory of a single fly walking in a circular open field arena. The fly was tracked for two hours. Here, we present techniques with which to analyze the motion of the fly in this paradigm, and we discuss the methods of calculation. The measures we introduce are based on spatial and temporal probability distributions and utilize the entire time-series trajectory of the fly, thus emphasizing the dynamic nature of locomotor behavior. Marginal and joint probability distributions of speed, position, segment duration, path curvature, and reorientation angle are examined and related to the observed behavior.

**Conclusions/Significance:**

The measures discussed in this paper provide a detailed profile of the behavior of a single fly and highlight the interaction of the fly with the environment. Such measures may serve as useful tools in any behavioral study in which the movement of a fly is an important variable and can be incorporated easily into many setups, facilitating high-throughput phenotypic characterization.

## Introduction

Characterizing the locomotor behavior of animals is essential to any study of the genotype-phenotype interaction. This is especially true for *Drosophila melanogaster*—genetic models of complex behaviors such as memory [1]–[4], drug addiction [5]–[7], and sleep [8]–[11], for example, are all based on measures that depend upon some observable movement of the flies. These observations typically take the form of general activity measures, based on the average speed of flies in some environment [6], [12], [13] or a simple line-crossing assay [8]–[11], [14], [15] (see the review by Martin [16] for a comprehensive discussion of locomotor studies in *Drosophila*). Because these methods can measure relative activities of populations, they lend themselves well to high-throughput assays and have been essential in uncovering genes involved and mechanisms behind some of these more complex behaviors.

The importance of locomotor activity, however, is often overlooked in and of itself as an important characteristic of the phenotype. Establishing a quantitative description of such behavior can help overcome any anthropomorphic bias of the investigator in inferring levels of fear, stress, anxiety, or attention. For this, however, it is advantageous to define measures that take into consideration the entire time series of movement of the fly—the trajectory that the fly takes during a given behavioral test. Because locomotor activity is a dynamic process, not only can analysis of such a trajectory provide measures of activity, but it can also shed light into the neurological and biochemical processes involved as the behavior unfolds over time. Such a study of the trajectory is best done in the Open Field, an environment which serves to approximate how animals would move in a natural setting.

Open-field behavior has been studied in mammals for some time [17]–[20] and in flies under other contexts (such as Buridan's paradigm [12], [21]), but has only recently been utilized as a valid assay for determining fly locomotor behavior [6], [7], [13], [16]. In this paper, we supplement the current metrics present in the literature for quantification of locomotor activity (see, for example, [18] and[13]) by presenting methods and metrics for analyzing the time-series trajectory of a fly walking in a circular, open-field arena. As an example of these methods, we analyze the trajectory of a single wild-type fly. While such single fly assays are open to the criticism of being time-consuming and tedious, it should be possible to improve throughput by studying multiple arenas at the same time, and multi-fly generalizations are clearly possible, though we will not address them in the current paper. Because only a single fly is studied in this paper, it should be noted that the results are not meant as generalizations of fly behavior. The emphasis in this paper, rather, is on the methods used for analysis of the trajectory.

The measures we introduce attempt to completely characterize the trajectory of a fly in the experimental environment, and can thus serve as the basis for a complete behavioral model of a walking fly. Performing such an analysis on multiple flies can refine such a model, and elucidate the individuality of phenotype under the control of constant genotype. The methods presented here utilize the entire time series obtained from tracking the fly over a long period of time, and can allow the experimenter to infer the influence of the environment on the resulting behavior—a point rarely considered in many behavioral assays.

## Methods

### Setup

**Figure 1:**
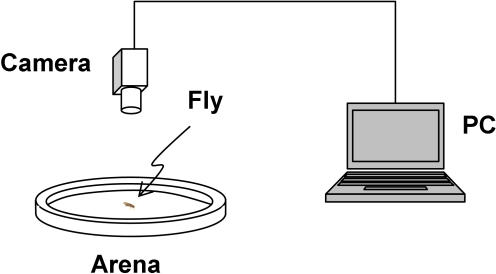
Experimental setup for characterization of locomotor behavior of *Drosophila* in the Open Field.

The experimental setup for observing a fly in the Open Field is relatively straightforward, and is shown in Figure 1. A single fly was placed in a circular arena 15 cm in diameter. A thin, transparent plastic ceiling was placed over the arena so that the fly did not escape during testing. Although this may influence the behavioral state, it eliminates any need for physical alteration of the fly, such as wing-clipping. The behavior of the fly was videotaped for a specified length of time at a frame rate of 25 Hz (40 ms time step). This sampling rate ensured that fast movements of the fly were sufficiently captured and allowed for a fine-grained analysis of the trajectory, yet was small enough for manageable file sizes. We have not yet established the ideal frame rate to capture fly movements, but it appears to be beneficial to go as high as 40 Hz. A region of interest in the captured video of size 720×560 pixels was saved directly to a computer for later analysis. For the example shown in this paper, two hours of behavior were videotaped and analyzed.

### Tracking

Following acquisition of the video, the position of the fly was tracked in each frame. For this study, the tracking was performed in the computing environment Matlab (http://www.mathworks.com/products/matlab/) using FTrack, a suite of functions and corresponding user interface written specifically for this research by one of the authors (DV). FTrack is available by request from the authors.

The tracking algorithm proceeds as follows [22]: In the videos acquired with the setup described above, the fly was a single ellipsoidal dot moving on a relatively constant background. Therefore, it was sufficient to find the fly in a frame obtained by subtracting the current frame **I**[*n*] from an averaged background **I** *_B_*[*n*] and squaring the result
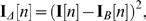
where *n* is the current frame index. The background image is calculated from a running average, following the rule
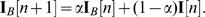
Here, 0.9<α<1 is a parameter that sets the (exponential) low pass temporal filter effectively used to compute the background. To further reduce false tracks, the area around the fly is excluded from the update, which prevents a motionless fly from becoming part of the background.

To determine the location of the fly, the pixel of maximum intensity is found in each frame, and a subset image **I**_Δ*sub*_ around this pixel is extracted. The center of intensity of the subset image is calculated and used to calculate the object's *x* and *y* locations
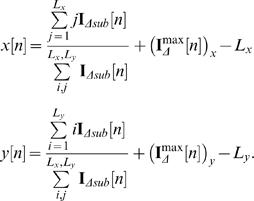
Here *i* and *j* correspond to row and column indices respectively; *L_x_* and *L_y_* are the dimensions of the subset image (in pixels); and 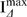 is the pixel of maximum intensity in the subset image. A caveat to the method is finding the fly in the first frame (*n* = 1). For this, a static background is calculated from a small number of initial frames. We have found that at a frame rate of 25 Hz, 100 frames is sufficient to calculate the background for a reasonably active fly.

### Correcting for Camera Tilt

**Figure 2:**
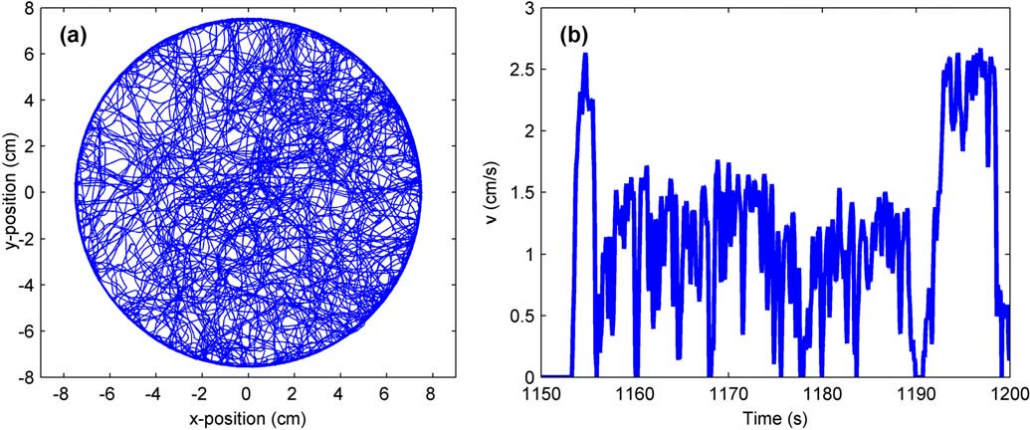
Position and speed characteristics of a fly moving in a circular Open Field arena. (a) The trajectory obtained from tracking a fly for two hours using the setup shown in Fig. 1. (b) The speed of the fly between 1150 and 1200 s, calculated by taking a numerical derivative of the smoothed position data. The stop-start behavior characteristic of fly locomotor activity is evident.

Although it is recommended to manually calibrate the orientation of the camera during the setup of the experiment, it is difficult to measure and completely eliminate any tilt that the camera plane may have. Unfortunately, such a tilt causes slight errors in measurement of positions and velocities from the video, since the video is capturing events projected onto a titled (as opposed to a parallel) plane. In the case of a circular arena, a tilt causes the arena to appear slightly elliptical. The deviation from circularity is measured by the eccentricity of the ellipse 
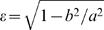
, where *b* is the length of the semiminor axis of the ellipse and *a* is the length of the semimajor axis. Thus, positions and velocities are stretched along the major axis of the ellipse. This becomes apparent upon examination of the spatial probability distribution in the case of a fly walking along the boundary walls (refer to Fig. 2 below); in polar coordinates, the location of the boundary will appear to oscillate around the arena radius instead of maintaining a constant value (figure not shown). Rotation of the camera confounds the issue.

Tilt and rotation of the camera can be corrected during post-processing. It can be shown that the eccentricity ε of the projected ellipse and the angle of tilt *ϕ* of the camera plane are related by the equation ε = *sin ϕ*. Therefore, to correct the trajectory data, points were selected manually around the arena boundary and an ellipse was fit to these points. The eccentricity and orientation (rotation) ψ of this ellipse were calculated and used to transform the data and back to a horizontal plane. This was done through the transformation equation
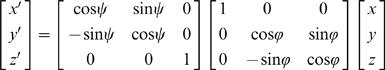
The trajectory projected into the (*x′,y′*) plane was taken as the transformed data. To confirm that the transformation was successful, points were selected around the boundary of the transformed trajectory and an ellipse was again fit. The eccentricity of this ellipse was zero, as to be expected for a circular arena.

### Trajectory Smoothing and Velocity Calculation

Due to noise and errors in tracking that occur for numerous reasons [22], [23], it is advantageous to smooth the resulting trajectory. This can be accomplished using standard moving-window polynomial regression techniques [23]–[25]. For this study, trajectories were smoothed using a running line regression with a window of 5 data points (0.2 s) and a 1 point step size (0.04 s) between windows. This step size corresponds to maximum overlap of the smoothing windows. The line regression was performed with the function ‘runline’ that is contained in the Chronux data analysis software package (currently implemented as a Matlab toolbox and available at http://www.chronux.org).

From smooth position trajectories, the velocity can then be calculated. There are two methods by which to do this. First, polynomial regression not only provides a smooth estimate of the position, but also allows a direct estimate of the velocity. The coefficient of the linear term of the local polynomial fit provides the velocity at that time step. An alternative, yet somewhat simpler, calculation of the velocity can be done by taking a numerical derivative of the position trajectory; however, one must ensure the trajectory has been properly smoothed if this method is chosen—the derivative of noise is undefined and can provide the researcher with meaningless velocity estimates. For this paper, the latter method was used. In any case, one should take care that both the position and velocity obtained after smoothing is physically plausible and corresponds to the actual movement seen in the video.

### Histograms and Density estimates

The trajectory of the fly, regarded as a spatial stochastic process **x**(t), would be fully characterized if the joint distributions P(**x**(t_1_),**x**(t_2_),…,**x**(t_n_)) were specified for all choices of time points (t_1_,t_2_,…,t_n_). Such probability functionals are widely used in statistical physics. The most general such process cannot be easily characterized, and one often resorts to simplified models such as Gaussian stochastic processes or Levy laws. Neither is particularly appropriate here: the position distributions are strongly non-Gaussian due to the influence of the arena, and the speed distributions are not consistent with Gaussian distributions. Furthermore, the departure from Gaussian behavior cannot be simply characterized by long tailed processes such as a Levy flight.

One approach would be to describe the trajectory using a Langevin style equation, commonly used to describe random walkers in statistical physics, and incorporate effects of the environment through boundary conditions. This is not a particularly useful approach, however, since the trajectories are smooth and proceed in smoothly curved segments, punctuated by stops.

Instead, we adopt another tool from statistical physics for characterizing processes by providing the joint distribution of the position and velocity at a given time, P(**x**,**v**). This is a marginal distribution of the full trajectory process, and therefore does not fully characterize the trajectory. However, it provides a convenient summary, and has the important advantage that it includes the effects of the environment explicitly. A less refined characterization would be to provide only the position distribution P(**x**), but it is clearly of interest to simultaneously consider the velocity distribution. In previous work, these two distributions have been considered separately. To our knowledge, this appears to be the first application of the joint position-velocity distribution for characterizing the trajectory. This characterization in terms of the joint position-velocity distribution also provides an alternative to arbitrary segmentations of the arena into center and wall zones for the arena.

We estimated the joint position-velocity distributions using a variety of histogram estimates. When examining histogram estimates of these probability distributions, one needs to exercise care about phase space factors in order to obtain accurate estimates. For example, because the fly is moving in two dimensions, the probability density for the speed *v* along with the phase space factors is given by *p(v)vdvdϕ* (where *ϕ* is polar angle of the point (*v_x_,v_y_*) in velocity space). Therefore, binning data in bins of size Δ*v*Δ*ϕ* would yield an estimate for *p(v)v*. In our estimates of *p(v)*, we eliminate the need to divide by *v* (which could be an unstable calculation for small *v*) by binning in *v* ^2^, since *p(v)vdvd̂∼p(v^2^/2)d(v* ^2^/2)*d̂*. Similar arguments can be made for the radial spatial distribution *p(r)*. One should take note of the non-constant bin widths on these histograms. For one-dimensional motion, such as movement along the arena boundary, there are no phase space factors and it is sufficient to bin the data in *v*.

### Segmentation of Velocity

Similar to many other animals [23], the locomotor behavior of flies is typically segmented into two (or more) regions of motion. Flies typically walk in a staccato manner, marked by bouts of fast walking episodes interspersed among brief stops or slowed speed [6], [13]. In the fly literature, these segments of differing speed are typically referred to as activity and inactivity and are defined by setting hard thresholds on the movement.

In the rodent literature, by contrast, much work has been done on this segmentation procedure to ensure reproducibility of tests across labs and across animals [26], [27]. Studies of the exploratory behavior of rodents have demonstrated that speed distributions provide a natural way to segment the motion. Instead of activity and inactivity bouts, mammalian locomotor studies define slow speed segments as lingering episodes and high speed segments as progression episodes. Complete stops are defined as arrests. In these studies, progression and lingering are defined through an examination of the average maximal speed distribution in segments between arrests. A threshold between progression and lingering is then determined by fitting Gaussian peaks to the resulting multimodal distribution.

For this paper, speed distributions are used to segment the motion where applicable; however, in contrast to [26] we do not plot the distribution of maximal speeds between arrests. Instead, the distribution *p(v)* is created from the *entire* speed time-series, which provides an alternative to what is typically seen in both the fly and rodent literatures. Using this measure, we define *near-zero speed* and *finite speed* motion, which do not necessarily correspond to activity, inactivity, progression, or lingering; it is merely a objective statement about the speed of the fly as it moves about the arena. *Stops* are defined as points in which the speed falls below the noise threshold of the tracking, which was determined from visual inspection of the velocity plots to be 0.1 cm/s. This threshold was chosen by noting the maximum measured velocity in segments of the video where the fly remained stationary to the eye of an observer. These points were then assigned zero velocity. While the method of segmentation based on a visual inspection of *p(v)* used here offers simplicity, it has yet to be examined in comparison to the more elaborate method of segmentation described in [26].

## Results

### Spatial Preference Distributions

Figure 2a shows a smoothed trajectory obtained from approximately two hours of video of a wild-type fly in the open field; a short section of the corresponding speed profile is presented in Fig. 2b. (Here, the characteristic staccato motion of the walking fly, described above, is evident.) The trajectory data exemplified in these two plots serve as a complete set from which all other locomotor parameters are inferred.

**Figure 3:**
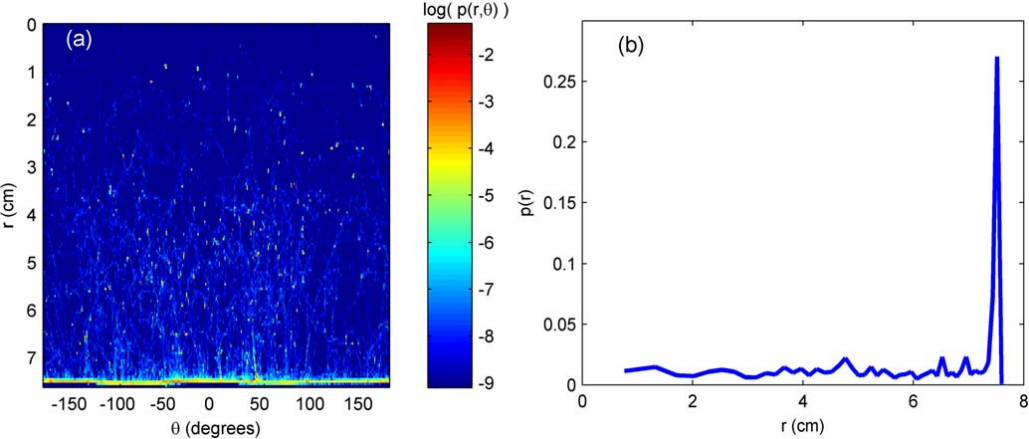
Spatial preference for a fly in the circular Open Field arena. (a) The joint probability distribution *p(r,^)* obtained using a bin size of 1° in ^, 0.05 cm in *r*. There appeared to be little angular preference, but the preference for the arena wall (*r* = 7.5 cm) is evident. (b) Marginal radial probability distribution *p(r)*. The histogram was obtained by binning *r^2^* in bins of size 1.13 cm^2^ (50 total bins). This estimate suggests defining a boundary between a Rim Zone and Central Zone at approximately *r* = 7.3 cm.

It is evident that the fly explored nearly the entire arena over the course of the two hours (Fig. 2a), and one immediately questions whether the fly displayed any spatial preference for one part of the arena. This can be determined by looking at the joint distribution of the fly's spatial location, *p(r,^)*, over the entire two hours (Fig. 3a). A transformation is made from Cartesian coordinates (*x,y*) to polar coordinates (*r,^*) since polar coordinates are more natural to the geometry of this environment. The origin is defined as the center of the arena. It is immediately clear that the fly prefers locations near the arena wall, spending 42% of its time within 1 cm of the wall, and the rest of the two hours distributed approximately equally throughout the arena. This conclusion is supported by examining the marginal distribution *p(r)* (Fig. 3b).

### Velocity Distributions and Segmentation of Behavior

**Figure 4:**
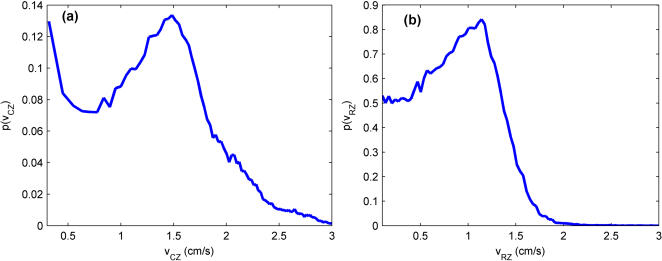
Speed distributions in each spatial ‘zone.’ (a) Speed distribution in the Central Zone, obtained using a bin size of 0.1 cm^2^ in *v* ^2^. Two visible behavioral components are seen in the plot, near-zero speed segments, marked by the tail descending from the zero speed bin, and finite speed segments, marked by the peak in the distribution at a non-zero, finite velocity (∼1.48 cm/s). A transition between these two segments can be estimated at about 0.75 cm/s. The first bin, which contains stops, is not shown in the figure. (b) Speed distribution in the Rim Zone, obtained using a bin size of 0.033 cm in *v.* Bins containing stops are not shown in the figure. Similar to the Central Zone distribution, this distribution has a peak at finite velocity (∼1.14 cm/s), but the near-zero segments do not show the large tail descending from zero seen in (a). A transition between the two segments in this zone can be estimated at about 0.4 cm/s.

The segmentation of the arena into spatial zones based on the trajectory data is clearly possible, but we have yet to determine if the fly moves differently in these two zones. To answer this question, we next examined the speed distribution *p(v*) in each zone (Fig. 4a and 4b). Note that the scales are different on each plot.

**Figure 5:**
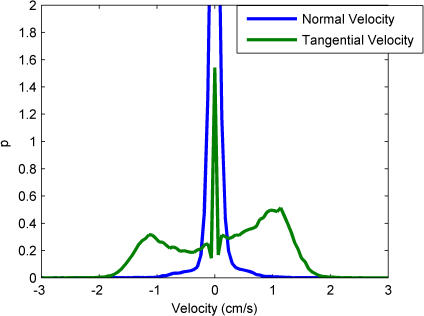
The probability distributions of velocities tangential to and normal to the wall in the Rim Zone. The fact that the normal (radial) velocity is concentrated near zero suggests that the Rim Zone motion should be treated as one-dimensional. The peak at zero for the normal velocity distribution reaches up to *p* = 5.93, and is eliminated for clarity.

Comparison of these distributions is not an entirely trivial exercise, since comparing motions of different dimensionality can lead to misleading conclusions about the distributions' shapes. We established above that in the Rim Zone, the fly walks mainly along the wall of the arena. When this is the case, the motion is approximately one-dimensional—the velocity of the fly should always be tangent to the wall. Examination of the velocity distributions for velocities tangent to and normal to the wall shows a normal velocity distribution that is narrowly peaked around zero, whereas the tangent velocity distribution is much broader (Fig. 5). Because of this, we do not need to account for the phase factor that arises in two dimensions and therefore bin the data in *v* instead of *v^2^* (See the section ‘Histograms and Density Estimates’). Note, therefore, that the estimates in Fig. 4a and 4b were obtained using different bin sizes.

Unfortunately, the differing bin sizes obfuscates the comparison of the distributions at low speeds. In Figure 4a, the distribution appears to fall exponentially from the noise threshold (0.1 cm/s), whereas in Fig. 4b, the low-speed distribution is essentially flat. It was difficult to determine whether these differences are inherent to the distributions or were an artifact from the binning procedure, and will be the subject of further study. Nonetheless, the speed distributions show similar behavior in that there are arguably two regions of motion, low-speed motion and finite-speed motion. Visual inspection suggests a speed threshold for the Central Zone at *v_th_* = 0.75 cm/s and for the Rim Zone at *v_th_* = 0.4 cm/s The segments with speeds below *v_th_* are defined as the *near-zero speed segments*; those above are the *finite speed segments*. The finite speed segments are marked by the peak in the distribution at a finite speed, approximately 1.48 cm/s in the Central Zone and 1.14 cm/s in the Rim Zone.

It is not trivial that these distributions show non-zero peaks. This suggests flies have a preferred walking speed, and this preferred speed depends on distance from the boundary in the environment. If the fly were a random-walker (Gaussian speed distribution), the only peak would occur at zero and nowhere else; a plot of log(*p*(*v^2^*)) would appear as a straight line of negative slope. Clearly, the trajectory cannot be described by a Gaussian stochastic process. Although this is not particularly surprising, it should be taken as a cautionary note before attempting to apply Gaussian autoregressive process models to such trajectory data.

**Figure 6:**
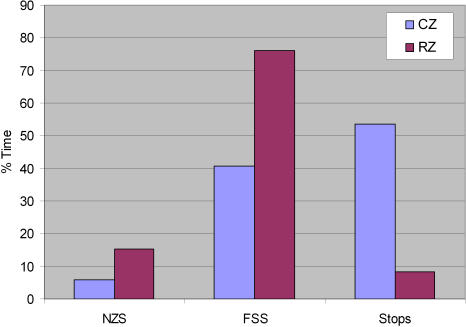
Percent time spent in different speed segments in each spatial zone. NZS – near-zero speed segments; FSS – finite speed segments; RZ – Rim Zone; CZ – Central Zone.

While the distributions are similar, the fly's speed behavior is quite different in the two zones. Figure 6 shows that more time is spent in motion in the Rim Zone than in the Central Zone (both in near-zero segments and finite speed segments); however, when in the Central Zone, the fly spends the majority of its time stationary. On the other hand, examination of the finite speed peaks (Fig. 4) shows that movement in the Central Zone occurs at a much faster preferred speed than does movement in the Rim Zone. There also appears to be several higher velocity bumps in the distribution of speed in the Central Zone that may correspond to different “gears” of progression that have been reported in rodent locomotion [26]. It is difficult to draw this conclusion, however, with only an N of one, and we present these data only to stress that the behavior does appear to be different in each zone. From these plots, in conjunction with Fig. 2, one may conclude that the fly spent the majority of its time exploring the rim of the arena at a slower typical speed, stopping infrequently. When it did venture away from the rim, the incursions through the Central Zone were at a faster typical speed. Clearly, the arena size and boundaries significantly influenced the animal's behavior.

**Figure 7:**
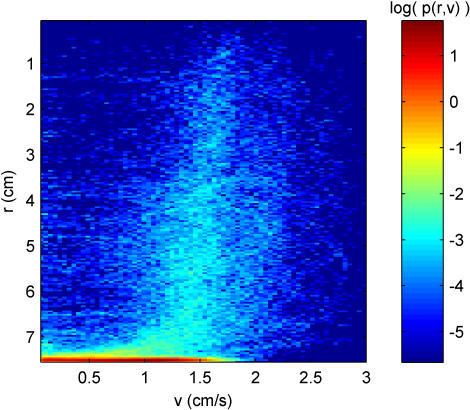
The joint probability distribution *p(r,v)* describing how fast the fly tends to walk in different parts of the arena. There is a slight increase in the finite speed peak location and narrowing of the distribution as the fly gets closer to the center of the arena (*r* = 0 cm).

The influence of the geometry on the behavior is best illustrated by the joint distribution *p(r,v)* (Fig. 7), which gives a measure of the speed of the fly in each radial portion of the arena. This distribution more clearly depicts the fine structure of the transition from Rim Zone to Central Zone behavior, as well as the fine structure of behaviors within the Central Zone. For example, one may argue that between *r* = 0 cm and 3.5 cm, the finite speed peak in the speed distribution is narrower compared to *r* in the range 3.5 cm to 7 cm. In words, the fly's speed is more stereotypical in the center of the arena than towards the edges of the arena. Clearly, marginal distributions obtained from the entire time-series trajectory can be used effectively to describe the behavior of a wild-type fly in the open field.

### Duration Distributions

**Figure 8:**
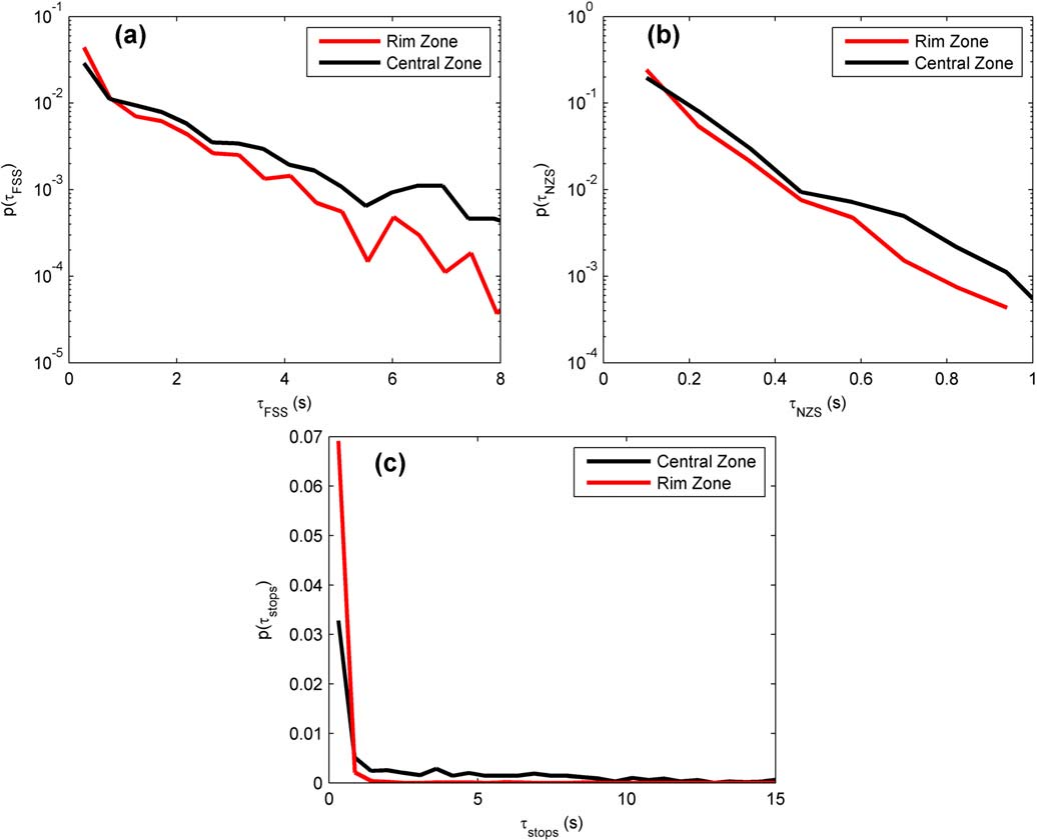
Comparison of duration distributions for (a) finite speed segments (FSS) (b) near-zero speed segments (NZS) and (c) stops in the Central Zone (black lines) and the Rim Zone (red lines). Note the different scales on the plots. Bin sizes are: (a) 0.48 s, (b) 0.12 s, and (c) 0.55 s.

**Figure 9:**
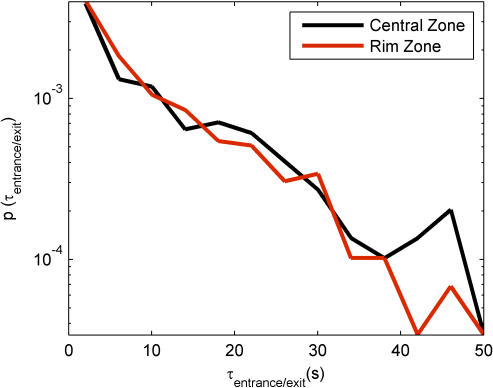
Distribution of the time between entrance into and exit from the Central Zone and the Rim Zone (bottom). This can also been interpreted as the “length of stay.” A bin size of 4 seconds has been used to bin the data.

The results of Figure 6 establish that the fly spends more time in motion in the Rim Zone and more time stopped in the Central Zone. This leads to the question of how long these behavioral segments typically last and whether the segment duration distributions differ between zones. The next trajectory measures examine this, and further characterize the behavior of the fly in the arena. These are the duration distributions for finite speed segments, near-zero speed segments, and stops (Fig. 8), and the distribution of entrance/exit times from the different zones (Fig. 9). Each of these distributions can be used to attach relevant time scales to the behavioral segments described above.

For both finite speed and near-zero speed segments, the duration distributions appear to exponentially decay (Figs. 8a and 8b, log scale). The majority of motion segments are fairly short; rarely did the fly spend more than 10 s in any behavioral segment (although there are a few instances when this did occur, and they mainly occurred in the Central Zone). In addition, behavior in the Central Zone and Rim Zone is similar for both types of motion, although the Rim Zone distributions have a slightly steeper decay (finite speed segments decay rates: 2.1 s in CZ and 1.37 s in RZ; near-zero speed segments: 0.14 s in CZ and 0.17 s in RZ). This suggests that Rim Zone motion segments are typically shorter than those in the Central Zone. Figure 8c shows the stop duration distribution for both zones. Stops in the Rim Zone are very short. Nearly 76% of the stops are less than 0.3 s, whereas stops in the Central Zone have a finite probability of being much longer (>5 s).

As seen in the figures, the main difference between finite speed segments and near-zero speed segments is that the near-zero speed segments occur on a much shorter time scale than the finite speed segments. This result may be attributed more to the fact that a finite speed segment is always proceeded and followed by a near-zero speed segment than to any outright behavioral aspect. That is to say: these data do not suggest that the fly is “choosing” to walk slowly for shorter lengths of time, but show the physical nature of the locomotor behavior. In fact, the near-zero segments provide one with a profile of the acceleration/deceleration ability of the fly. This acceleration and deceleration occurs on very vast time scales (<1 s)—the fly does not slowly work its way up to full speed, nor to a stop.

One can also examine the distribution of the events marked by the entrance into and the exit from each zone, which can yield a measure of likelihood that the fly remains in that particular zone. These are shown in Fig. 9. The distributions for the events are quite similar for both zones, showing that the fly rarely stays in a particular zone for longer than a minute. Although Fig. 2 implies that the fly prefers the Rim Zone, Fig. 9 implies that the length of stay in each zone is relatively the same. This is not a contradiction—note that the area of the Central Zone is larger than that of the Rim Zone, and so segments of equal duration (both stop and motion segments) are distributed over a larger area. In fact, the fly spends more *total* time distributed throughout the Central Zone (see Fig. 2), but the Rim Zone is a more confined area, and the probability density is higher for finding the fly at a location in the Rim Zone compared to a location in the Central Zone .

The data presented above imply behavior in which the fly is more likely to linger, yet takes longer, faster excursions when it chooses to walk in the Central Zone. In contrast, the fly has a slower, more staccato-type walk in the Rim Zone, perhaps suggesting a behavior more exploratory in nature.

### Path Curvature and Reorientation Angle

**Figure 10:**
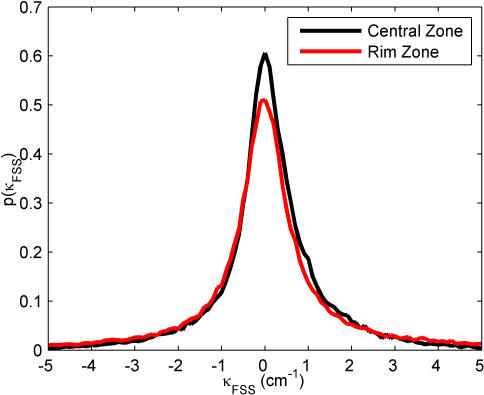
Distribution of path curvature during finite speed segments in the Central Zone (black line) and the Rim Zone (red line). Bin size: 0.1 cm^−1^. Both distributions are peaked around κ = 0, which corresponds to movement in a straight line.

**Figure 11:**
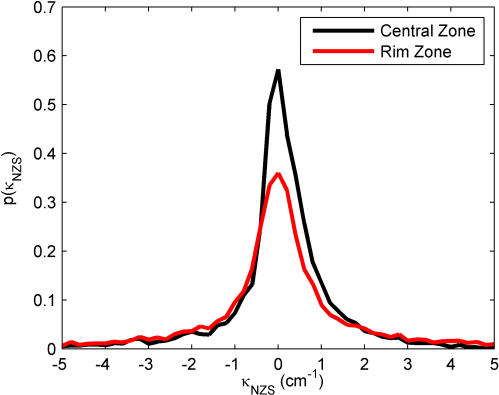
Distribution of path curvature during near-zero speed segments in the Central Zone (black line) and the Rim Zone (red line). Bin size: 0.2 cm^−1^.

Two more quantities can be used to characterize the locomotor behavior of the fly in the open field: path curvature and reorientation angle. The curvature of a path is defined as
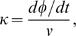
where φ is angle of the velocity vector and *v* is the speed. The distribution of κ for each zone is shown in Fig. 10 for finite speed segments and Fig. 11 for near-zero speed segments; κ = 0 corresponds to a straight-line trajectory. For both zones and speed behaviors, the distributions are peaked at zero curvature and have relatively symmetric distributions, the exception being near-zero motion in the Central Zone, which shows a slight skewness towards counter-clockwise motion (κ>0). It is interesting to note that the wall does not appear to affect the path curvature; there are no visible peaks at ±0.13 cm^−1^, the curvature of the arena. However, this is close to the bin size in our computation, and a more tightly curved arena might produce corresponding peaks in the curvature distribution in the rim zone.

**Figure 12:**
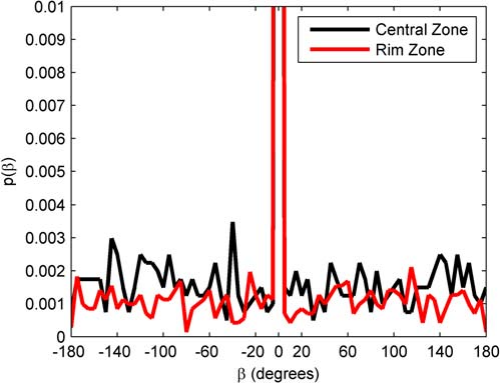
Distribution of reorientation angle, β, in the Central Zone (black line) and the Rim Zone (red line). In both zones, the fly prefers to walk in the same direction after a stop as it was heading before the stop. Because the peak at zero is so large for both zones (>0.1), the plot has been truncated to better display the characteristics of the distribution. Bin size: 5 degrees.

Finally, one can examine the tendency of the fly to change direction after stops, a similar measure to those defined for chemotaxis and thermotaxis in *E. coli* [29] and *C. elegans* [30], [31]. We define this as the *reorientation angle* β; it is shown in Fig. 12. After these segments, the fly appears to move in the same direction as the last frame before the arrest in the majority of instances (zero reorientation). In the cases where the fly does change its direction, the choice of direction appears to be uniformly distributed throughout angular space. Whether this is the influence of the environment or hallmark of some internal motivation is not entirely evident.

## Discussion

In order to complement the metrics that are currently used to describe the locomotor behavior of *Drosophila* (average activity and inactivity, bout length, distance moved, turning angle, etc.) [6], [12]–[15], we have presented measures that utilize the entire trajectory of the walking fly in a circular arena. The insight that can be obtained by a time-series analysis is often underappreciated, perhaps due to the lack of methods with which to handle the large amounts of data that a trajectory provides. However, metrics obtained using time-series analysis methods have already been highly beneficial in the field of neuroethology [32].

It is clear that locomotor activity is greatly influenced by the environment within which the behavior occurs. Such an interaction can in general be difficult to characterize, and one quickly realizes that by observing an animal in an environment, as much is learned about the structure of the environment as is learned about the behavior of the animal. Given Fig. 2, one does not have to know any details of the setup to infer that the fly was restricted to a circular arena. By examining the joint probability distributions *p(r,^)* and *p(r,v)*, the curvature of the path, and the reorientation angle, we obtain measures that directly account for the effects of the geometry of the arena on the locomotor trajectory. Along with the duration distributions, these measures should allow for the construction of realistic trajectory models in the open field. They also promise to provide quantitative phenotypic measures for *Drosophila* locomotion under circumstances which exhibit greater ethological realism.
